# Social Network Types and Health Among Older Singaporeans

**DOI:** 10.1093/geroni/igaa057.1311

**Published:** 2020-12-16

**Authors:** Rahul Malhotra, Pildoo Sung, Angelique W M Chan

**Affiliations:** Duke-NUS Medical School, Singapore, Singapore

## Abstract

Little is known about the heterogeneity and dynamics in older adults’ social networks and their bidirectional relationship with health in Asian societies. We investigate (1) social network types, (2) how network types predict health, and (3) whether health influences network types over time among older Singaporeans. We use data from Transitions in Health, Employment, Social engagement and Inter-Generational transfers in Singapore Study (THE SIGNS Study), a national longitudinal survey, collected in 2016-2017 (wave 1) and 2019 (wave 2). Latent class analysis is applied to identify distinct social network types and how they affect self-rated health after two years. Latent transition analysis is then employed to examine the pattern of change in network types between waves, and the relationship between baseline self-rated health and transition in network types. We identify six social network types: diverse, diverse but less socially engaged, immediate family, extended family, living alone yet diverse, and restricted (proportion at baseline: 7.2 %, 38.2 %, 14.1 %, 27.1 %, 7.0 %, and 6.4 %, respectively). Older adults in the ‘living alone yet diverse’ network type are less likely to report poor self-rated health after two years than those in the restricted and extended family network types. Additionally, we find that good health is related to more diversified network types—‘diverse’ and ‘diverse but less socially engaged’—at baseline, and network types are relatively stable over two years. These findings contribute to the literature by capturing complexities in the reciprocal relationship between social network types and health in later life.

